# Optimizing Expectations About Endocrine Treatment for Breast Cancer: Results of the Randomized Controlled PSY-BREAST Trial

**DOI:** 10.32872/cpe.v2i1.2695

**Published:** 2020-03-31

**Authors:** Meike C. Shedden-Mora, Yiqi Pan, Sarah R. Heisig, Pia von Blanckenburg, Winfried Rief, Isabell Witzel, Ute-Susann Albert, Yvonne Nestoriuc

**Affiliations:** a Department of Psychosomatic Medicine and Psychotherapy, University Medical Center Hamburg-Eppendorf, Hamburg, Germany; b Department of Clinical Psychology and Psychotherapy, Hamburg University, Hamburg, Germany; c Department of Clinical Psychology and Psychotherapy, Philipps-University of Marburg, Marburg, Germany; d Department of Gynecology, University Medical Center Hamburg-Eppendorf, Hamburg, Germany; e Department of Gynecology, University Medical Center Würzburg, Würzburg, Germany; f Department of Clinical Psychology, Helmut Schmidt University Hamburg, Hamburg, Germany; Philipps-University of Marburg, Marburg, Germany

**Keywords:** expectation management, nocebo effect, psychological intervention, side effect, adjuvant endocrine treatment, breast cancer, oncology

## Abstract

**Background:**

Medication side effects are strongly determined by non-pharmacological, nocebo mechanisms, particularly patients’ expectations. Optimizing expectations could minimize side effect burden. This study evaluated whether brief psychological expectation management training (EXPECT) optimizes medication-related expectations in women starting adjuvant endocrine therapy (AET) for breast cancer.

**Method:**

In a multisite randomized controlled design, 197 women were randomized to EXPECT, supportive therapy (SUPPORT), or treatment as usual (TAU). The three-session cognitive-behavioral EXPECT employs psychoeducation, guided imagery, and side effect management training. Outcomes were necessity-concern beliefs about AET, expected side effects, expected coping ability, treatment control expectations, and adherence intention.

**Results:**

Both interventions were well accepted and feasible. Patients’ necessity-concern beliefs were optimized in EXPECT compared to both TAU and SUPPORT, d = .41, p < .001; d = .40, p < .001. Expected coping ability and treatment control expectations were optimized compared to TAU, d = .35, p = .02; d = .42, p < 001, but not to SUPPORT. Adherence intention was optimized compared to SUPPORT, d = .29, p = .02, but not to TAU. Expected side effects did not change significantly.

**Conclusion:**

Expectation management effectively and partly specifically (compared to SUPPORT) modified medication-related expectations in women starting AET. Given the influence of expectations on long-term treatment outcome, psychological interventions like EXPECT might provide potential pathways to reduce side effect burden and improve quality of life during medication intake.

## Abstract (highlights)

- Expectation management (EXPECT) optimizes expectations prior to endocrine therapy for breast cancer.
- EXPECT improved necessity-concern beliefs, coping and control expectations and adherence intention.
- EXPECT was partly more effective than the supportive therapy control condition.
- Expectation management provides a pathway to reduce side effect burden during long-term medication.

Medication side effects are substantially determined by mechanisms which are not directly attributable to the pharmacodynamics of the treatment. These non-specific side effects are well-known from the nocebo phenomenon which manifests itself when adverse effects occur after placebo intake (Barsky, Saintfort, Rogers, & Borus, 2002). Nocebo effects may also emerge as part of routine treatments. Hence, non-specific medication side effects might aggravate the impact of specific side effects (Rief, Bingel, Schedlowski, & Enck, 2011).

Nocebo-related side effects are predominantly determined by psychological mechanisms, most relevantly patients’ expectations (Webster, Weinman, & Rubin, 2016). Expectations are influenced by treatment information, social observation, and other learning processes through negative experiences with prior medication intake (Colloca & Miller, 2011). Analogous to expecting treatment benefits, patients also develop expectations about potential adverse events (Laferton, Kube, Salzmann, Auer, & Shedden-Mora, 2017), and form beliefs about their medication’s necessity and possible concerns (Horne, Weinman, & Hankins, 1999). These side effect expectations and medication beliefs are linked to the actual occurrence of side effects of cancer treatments (Colagiuri & Zachariae, 2010; Nestoriuc et al., 2016), and other therapies (Faasse & Petrie, 2013; Nestoriuc, Orav, Liang, Horne, & Barsky, 2010). Importantly, side effect expectations and medication beliefs not only predict long-term quality of life, but also medication non-adherence (Horne et al., 2013; Nestoriuc et al., 2016; Pan et al., 2018).

As expectations are potentially modifiable factors, optimizing patients’ treatment expectations has been put forward as a novel strategy to improve treatment outcome and minimize side effect burden (Bingel, 2014; Heisig, Shedden-Mora, Hidalgo, & Nestoriuc, 2015; Laferton et al., 2017; Nestoriuc et al., 2016). First evidence from experimental studies suggests that psychological expectation management can effectively improve participants’ expectations regarding anti-cancer treatments (Heisig, Shedden-Mora, Hidalgo, & Nestoriuc, 2015), reduce pain (Peerdeman et al., 2016) and even reverse nocebo effects (Bartels et al., 2017). To date, the PSY-HEART-trial (Rief et al., 2017) showed that brief expectation management prior to open-heart surgery successfully changes expectations (Laferton, Auer, Shedden-Mora, Moosdorf, & Rief, 2016), improves long-term disability, quality of life and reduces the length of hospital stay (Auer et al., 2017).

This study employs expectation management in patients undergoing adjuvant endocrine therapy (AET) for breast cancer. AET is the state-of-the-art treatment for hormone-receptor-positive breast cancer. Intake for at least five years improves disease-free survival and time to recurrence (Burstein et al., 2014). Despite its proven clinical efficacy, non-adherence rates ranging from 28% to 73% within the 5-year intake period have been reported (Murphy, Bartholomew, Carpentier, Bluethmann, & Vernon, 2012). As low adherence is associated with poorer survival (Hershman et al., 2011), ensuring patients’ adherence is crucial. Side effects such as arthralgia, hot flushes, weight gain, and loss of libido can substantially reduce quality of life (Cella & Fallowfield, 2008) and cause treatment discontinuation (Demissie, Silliman, & Lash, 2001). Side effects occur related to the specific pharmacodynamics of AET (e.g., hot flushes are caused by the deprivation of estrogen), but can also be treatment-unrelated (e.g., dizziness) (Gibson, Lawrence, Dawson, & Bliss, 2009). Relevantly, side effect expectations predict the actual occurrence of cancer treatment side effects (Colagiuri & Zachariae, 2010), long-term quality of life, and non-adherence in AET (Nestoriuc et al., 2016; Pan et al., 2018).

The aim of this study was to evaluate whether a three-session psychological expectation management training (EXPECT; von Blanckenburg, Schuricht, Albert, Rief, & Nestoriuc, 2013; von Blanckenburg et al., 2015) optimizes patients’ AET-related expectations when starting AET. This study reports the pre- to post-intervention change of expectations of the PSY-BREAST trial (expectation-focused PSYchological pre-treatment intervention to improve outcome in BREAST cancer treatment). EXPECT was compared to a psychological control intervention (supportive therapy, SUPPORT), and treatment as usual (TAU). It is hypothesized that EXPECT but not SUPPORT and TAU improves expectations regarding the prescribed AET medication and its side effects, the expected ability to cope with potential side effects, and treatment control expectations. Secondly, it is hypothesized that only EXPECT improves the intention to adhere to AET.

## Method

### Study Design

**Figure 1:**
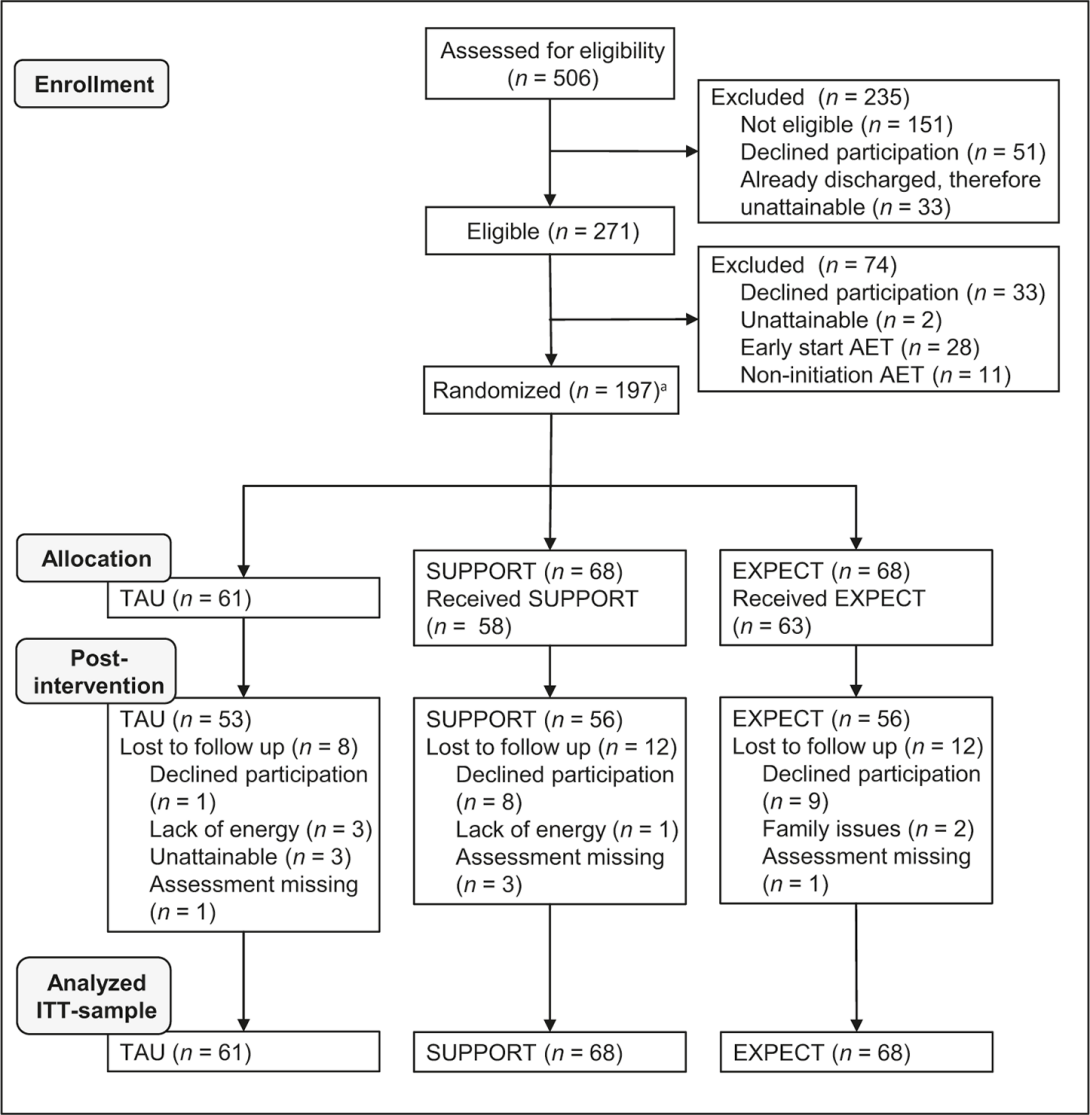
Patient Flow (CONSORT) *Note.* AET = adjuvant endocrine treatment; TAU = treatment as usual; SUPPORT = supportive therapy; EXPECT = expectation management training. ^a^Of *n* = 203 randomized patients, 6 were identified as non-eligible post-randomization and therefore excluded.

This was a three-arm multisite (two centers with four clinics), randomized controlled trial. It was registered at ClinicalTrials.gov (NCT01741883). Ethical approval was obtained from the respective local ethics committees (Marburg, Hamburg). Outcomes for this analysis were compared between baseline and post-intervention (Figure 1). A detailed description of the design is provided in the study protocol (von Blanckenburg et al., 2013). After study inclusion, patients were randomly assigned to receive EXPECT, SUPPORT, or TAU. Treatment as usual (TAU) in all groups consisted of the general guideline-based oncologic regime in the certified breast cancer centers, usually surgery and radiation, followed by adjuvant endocrine treatment with tamoxifen or third-generation aromatase inhibitors (Kreienberg et al., 2012). The decision of the type of AET mainly depended on the women’s menopausal status. All patients were offered one session basic psycho-oncological support by a trained psycho-oncologist of the hospital staff. After discharge, patients were treated in an outpatient setting by a gynecologist, general practitioner, and if desired, a psycho-oncologist with up to 12 sessions. Patients were allocated in a 1:1:1 ratio stratified according to the Hospital Anxiety and Depression Scale (sum score ≤13 vs. >13) and type of medication (aromatase inhibitor vs. tamoxifen).

### Participant Enrollment

Data were collected between November 2012 and May 2015 at the Philipps University of Marburg and the University Medical Center Hamburg-Eppendorf, Germany. Patients were recruited post-surgery during their hospital stay. Included were women aged 18-80 years, with hormone-receptor-positive breast cancer or ductal carcinoma in situ to whom first-line adjuvant endocrine treatment with tamoxifen or third generation aromatase inhibitors was prescribed. Further inclusion criteria were the ability to give informed consent and sufficient German language skills. Exclusion criteria were advanced breast cancer, the presence of any other cancer or comorbid somatic illness causing predominant disability, severe psychiatric illness (e.g., psychosis, checked by structured psychiatric interview, mini-DIPS), and adjuvant chemotherapy.

After providing written informed consent, all patients received a medication information leaflet accompanied by an oral briefing by trained research assistants. This previously validated information illustrated the mode of action, the desired effects, and potential side effects of AET in order to homogenize knowledge (Heisig, Shedden-Mora, von Blanckenburg, et al., 2015). The information briefing was followed by baseline assessment and randomization. Outcome assessors (trained research assistants) were blinded to group allocation throughout the study. For this analysis, the sample of *n* = 197 patients will allow the detection of small effect sizes, *f*(V) = .11, with 80% power and α = .05.

### Psychological Interventions

Patients received three individual weekly or bi-weekly treatment sessions of 50-75-minutes by a clinical psychologist, followed by up to three 15-minutes booster phone calls at one, three, and six months. A detailed description of the interventions can be found in the study protocol (von Blanckenburg et al., 2013) and case report (von Blanckenburg et al., 2015). All therapists received regular supervision by experienced psycho-oncologists. Therapist allegiance evaluated via video ratings was considered as high (Appendix).

#### EXPECT – Expectation Management Training

EXPECT is based on cognitive-behavioral therapy and aims to prevent nocebo-related side effects from AET by optimizing treatment-related expectations. The focus on side effects is counterbalanced by therapeutic work towards strengthening beliefs of treatment control, benefit, and necessity. EXPECT is manualized; however, topics are adapted to the patient’s individual expectations using a personalized intervention booklet. The three sessions have the following goals and topics:

*Session 1*. Psychoeducation about AET (mode of action, benefits, potential side effects) is given. The impact of expectations and the nocebo effect are discussed. The aim is to strengthen beliefs about AET’s necessity while keeping concerns at a realistic minimum (Heisig, Shedden-Mora, von Blanckenburg, et al., 2015). An imagery exercise guides the patients towards visualizing the expected benefits of AET.

*Session 2*. Coping strategies for managing the three individually most feared side effects are developed (Mann et al., 2012). These include behavioral techniques, cognitive strategies, and management of specific triggers. Strategies are summarized in a written problem-solving scheme, and patients are encouraged to create a practical ‘tool-box’.

*Session 3*. To strengthen resources for the medication intake period, resourceful activities (e.g., gardening) are encouraged. To support defocusing from side effects, attention control strategies are discussed. To enhance effective patient-doctor communication, patients receive a communication skills training. At the end of the session, the tool-box and all previous topics are reviewed.

*Booster calls*. The three booster calls aim to provide therapeutic support during the first months of medication intake. Patients are encouraged to apply the learned coping strategies for side effects, which are adapted if necessary.

#### Supportive Therapy (SUPPORT)

Supportive therapy was designed as an active psychological control condition to account for general therapeutic factors such as the therapist’s attention and the patient-therapist relationship (Markowitz, Manber, & Rosen, 2008). It allows distinguishing specific effects of EXPECT from psychological placebo effects. It applies common factors of psychotherapy such as elicitation of affect, empathy, and reflective listening. In contrast to EXPECT, no explicit theoretical framework and no expectation-targeted interventions are provided. Each session is structured into three phases: the beginning (inquiring about relevant topics), the therapeutic dialog (encouraging the patient to talk about any theme of affective valence), and the ending (revising addressed themes). The booster calls are conducted analogously to EXPECT, with focus on the patient’s emotional state.

### Assessment

#### Patients’ Expectations

Medication-related expectations about AET were assessed using the Necessity-Concern Balance as measured by the Beliefs about Medicines Questionnaire (BMQ; Horne et al., 1999). A difference score ranging from -4 to 4 is calculated by subtracting the mean expected necessity scale (5 items) from the mean expected concerns scale (6 items) (Horne et al., 2013). Positive scores indicate stronger necessity beliefs than concerns (≈ functional balance).

The mean intensity of 44 expected side effects was assessed using the General Assessment of Expected Side Effects Scale (GASE-expect; Nestoriuc et al., 2016) which measures the expected intensity of 23 general and 21 AET-specific side effects on a 0 (‘not present’) to 3 (‘severe’) scale.

The expected ability to cope with the potential 44 expected side effects in case of their presence was assessed on a 1 (‘expect to cope badly’) to 4 (‘expect to cope very well’) scale.

Treatment control expectation was assessed with the respective item (‘How much do you think your AET can help your breast cancer?’) from the Brief Illness Perception Questionnaire (B-IPQ), ranging from 0 (‘not at all’) to 10 (‘extremely helpful’). (Broadbent, Petrie, Main, & Weinman, 2006)

#### Adherence Intention

Adherence intention was assessed with the question ‘How certain are you about starting the endocrine therapy?’ rated on a 7-point scale (from 1 ‘very unsure’ to 7 ‘very sure’).

#### Sociodemographic and Medical Variables

Age, education, and marital status were assessed. Medical variables, namely menopausal status, and breast cancer tumor stage were retrieved from the hospitals’ patient records. Patients provided information on their prescribed AET and existing medical comorbidities. The presence and intensity of 44 current somatic complaints were assessed on a 0 to 3 scale using the GASE (Rief, Barsky, et al., 2011).

#### Patients’ Evaluation of the Intervention

Patients evaluated the intervention on nine statements rated from 1 (‘do not agree at all’) to 6 (‘fully agree’). The general satisfaction with the intervention, specific components of EXPECT, and therapeutic components imminent to supportive therapy were assessed. Potential adverse events of the interventions were assessed with an open-ended question.

Treatment fidelity was assessed by asking patients how often they practiced the imagery exercise on one 1 (‘daily’) to 5 (‘not at all’) scaled item. Additionally, participation in booster sessions was assessed.

Therapeutic alliance was rated by patients and therapists after each session with two questions (the intervention has helped me / the patient, the psycho-oncologist understands me / the patient felt understood) from 1 (‘do not agree at all’) to 6 (‘fully agree’).

### Data Analysis

To examine whether EXPECT resulted in improved expectations compared to SUPPORT and TAU, we computed linear mixed models with treatment group, time (pre- vs. post-intervention) and treatment group by time as fixed effects and a random intercept for subject-specific effects with a restricted maximum likelihood estimation and an autoregressive residual matrix. All analyses were adjusted for study site, age, type of AET, breast cancer tumor stage, and physical symptoms (GASE) as fixed effects. For the hypothesized treatment group by time interaction, pairwise comparisons were reported. Pre-post-tests were performed to indicate improvements within a group.

Missing values on single items ranged from 0 to 3.5% and were imputed using the EM-algorithm. Missing data points at post-intervention were estimated within the linear mixed model using the full intention-to-treat sample. Effect sizes were calculated as differences in mean growth rates between the groups, divided by the product of standard error by square rooted number of participants in TAU (Feingold, 2009). Significance level for all analyses was set at α = .05. Statistical analyses were performed using SPSS Statistics 24.

## Results

### Participant Flow

Of 506 women assessed for eligibility, 271 were eligible for study participation, 197 patients were randomized analyzed as the ITT-sample (Figure 1). Of those, 165 completed post-intervention assessment (83.8%).

Women who discontinued AET before post-intervention assessment (EXPECT: *n* = 0; SUPPORT: *n* = 4; TAU: *n* = 2), and women who did not start the intervention (EXPECT: *n =* 5, SUPPORT: *n =* 10), but completed post-assessment were included in the analyses to avoid selection bias. Fifty-four women (79.4%) in EXPECT and 55 women (80.9%) in SUPPORT received all three sessions.

### Baseline Characteristics

All baseline sociodemographic and clinical characteristics were comparable across the groups (Table 1).

**Table 1:** Demographic and Clinical Sample Characteristics

Variable	EXPECT (*n* = 68)	SUPPORT (*n* = 68)	TAU (*n* = 61)	Comparison	
				*F* | χ^2^	*p*
Demographics					
Age in years, *M* (*SD*)	56.46 (8.92)	58.44 (8.40)	59.64 (10.74)	*F*(2, 197) = 1.92	.15
At least 13 years of education, *n* (%)	24 (35.8)	28 (41.8)	21 (34.4)	χ^2^(2) = 0.85	.65
Married/with partner, *n* (%)	42 (61.8)	45 (66.2)	36 (59)	χ^2^(2) = 0.72	.70
Clinical symptoms					
Peri-/Post-menopausal, *n* (%)	49 (72.1)	53 (77.9)	48 (78.7)	χ^2^(2) = 0.96	.62
Tumor stage UICC, *n* (%)				χ^2^(4) = 3.49	.48
I	41 (60.3)	42 (61.8)	44 (72.1)		
II	23 (33.8)	24 (33.8)	16 (26.2)		
III	4 (5.9)	2 (2.9)	1 (1.6)		
Type of AET, *n* (%)				χ^2^(2) = 2.19	.34
Tamoxifen	37 (54.4)	35 (51.5)	39 (63.9)		
Aromatase Inhibitors	31 (45.6)	33 (48.5)	22 (36.1)		
Medical comorbidities, *n* (%)				χ^2^(4) = 0.76	.94
0	25 (36.8)	23 (33.8)	19 (31.1)		
1 or 2	35 (51.5)	38 (55.9)	36 (59)		
≥ 3	8 (11.8)	7 (10.3)	6 (9.8)		
Number of current somatic complaints (GASE)					
*M* (*SD*)	11.10 (6.70)	9.34 (6.20)	9.98 (7.11)	*F*(2, 197) = 1.22	.30
Range	0 - 31	0 - 26	0 - 29		
Intensity of current somatic complaints (GASE)					
*M* (*SD*)	0.33 (0.24)	0.30 (0.25)	0.31 (0.25)	*F*(2, 197) = 0.32	.73
Range	0 - 3	0 - 3	0 - 3		

*Note.* EXPECT = expectation management training; SUPPORT = supportive therapy; TAU = treatment as usual; AET = Adjuvant endocrine therapy; UICC = Union for International Cancer Control; GASE = General Assessment of Side Effects Scale.

The majority of the women were diagnosed with tumor stage I (64.5%). The most frequent comorbidities were hypertension (32.0%), thyroid diseases (25.9%), and joint or dorsal pain (18.3%). Most common baseline somatic symptoms comprised pain or sensitivity of the breast (71.6%), sleeping problems (52.3%), and fatigue (50.5%).

### Changes in Patients’ Expectations

The *necessity-concern balance* at baseline was rather positive in all groups (Table 2).

**Table 2:** Outcome Measures at Baseline and Post-Intervention

Outcome	EXPECT	SUPPORT	TAU	EXPECT vs. TAU			EXPECT vs. SUPPORT		
				*t*	*p*	*d*	*t*	*p*	*d*
Medication beliefs: necessity-concern balance (BMQ; range -4-4)				3.33	< .001	0.43	3.15	< .001	0.40
Baseline	0.68 [0.43, 0.93]	0.82 [0.57, 1.06]	0.77 [0.51, 1.04]		
Post-intervention	1.06 [0.79, 1.33]	0.63 [0.36, 0.90]	0.54 [0.27, 0.83]		
Expected side effects, mean intensity (GASE-expect; range 0-3)				-1.69	.092	-0.22	-0.66	.51	-0.09
Baseline	0.56 [0.48, 0.64]	0.50 [0.42, 0.59]	0.47 [0.38, 0.55]		
Post-intervention	0.53 [0.44, 0.62]	0.51 [0.42, 0.60]	0.54 [0.45, 0.63]		
Expected coping ability, mean (GASE coping; range 1-4)^a^				2.45	.015	0.35	1.44	.15	0.21
Baseline	3.49 [3.39, 3.58]	3.53 [3.44, 3.63]	3.61 [3.51, 3.72]		
Post-intervention	3.63 [3.53, 3.74]	3.56 [3.46, 3.66]	3.55 [3.44, 3.66]		
Expected treatment control (B-IPQ; range 0-10)				3.27	< .001	0.42	1.65	.10	0.21
Baseline	7.43 [6.89, 7.98]	7.51 [6.97, 8.05]	7.91 [7.33, 8.48]		
Post-intervention	7.73 [7.14, 8.31]	7.11 [6.53, 7.69]	6.79 [6.18, 7.40]		
Adherence intention (range 1-7)				1.85	.065	0.24	2.27	.024	0.29
Baseline	6.05 [5.73, 6.37]	6.37 [6.05, 6.69]	6.32 [5.98, 6.66]		
Post-intervention	6.66 [6.30, 7.01]	6.27 [5.91, 6.62]	6.33 [5.97 -6.70]		

*Note.* Values indicate estimated marginal means [95% CI]. Analyses are adjusted for study side, age, type of AET, breast cancer tumor stage, and baseline physical symptoms. Statistical comparisons (*t*- and *p*-values) refer to the pairwise comparisons of the treatment group by time interaction. EXPECT = expectation management training; SUPPORT = supportive therapy; TAU = treatment as usual.

^a^Sample size for analysis *n* = 172 (25 patients did not expect any side effects).

**Figure 2:**
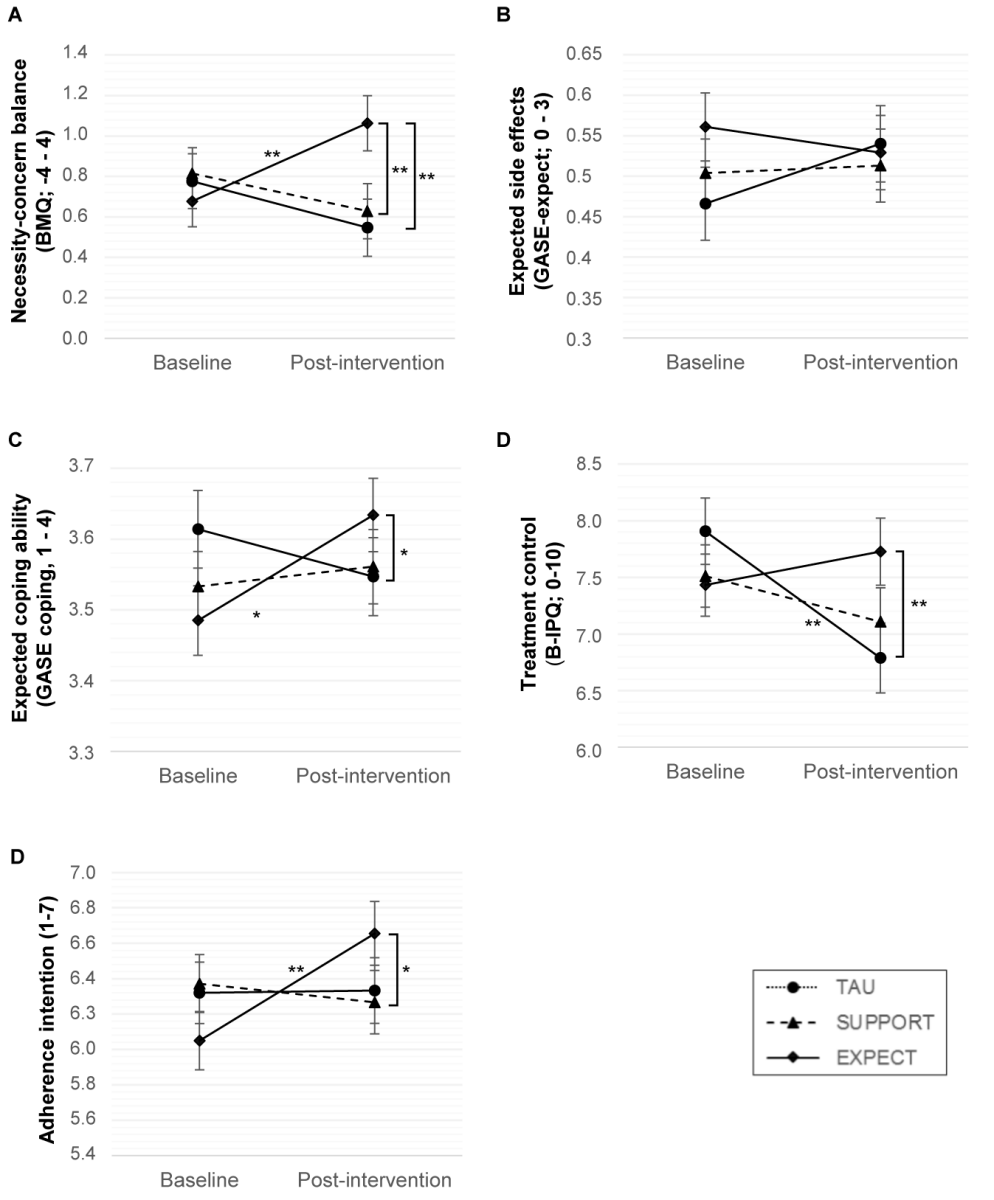
Expectations at Baseline and Post-Intervention *Note.* Values shown are estimated marginal means (error bars: ± 1 standard error) from linear mixed models. TAU = treatment as usual, SUPPORT = supportive therapy, EXPECT = expectation management training. Numbers after scale names indicate the range. *p < .05. **p < .01

A significant group by time interaction indicated an improved necessity-concern balance in EXPECT compared to both TAU and SUPPORT, estimated mean difference = 0.61, 95% CI [0.25, 0.98], *p* = .001; 0.57, 95% CI [0.21, 0.93], *p* = .002, respectively (Figure 2).

Pre-post within-group comparisons indicated that significant improvements in the necessity-concern balance only occurred in EXPECT but not in TAU and SUPPORT, 0.38, 95% CI [0.13, 0.64], *p* = .003; -0.23, 95% CI [-0.49, 0.03], *p* = .085; -0.19, 95% CI [-0.44, 0.07], *p* = .147. When the scales were analyzed separately, EXPECT showed an increase of necessity beliefs compared to SUPPORT and in trend to TAU, 0.27, 95% CI [0.00, 0.54], *p* = .049; 0.25, 95% CI [-0.03, 0.53], *p* = .075, respectively. EXPECT reported a reduction of concerns compared to TAU and SUPPORT, -.37, 95% CI [-.59, -.14], *p* = .002; -.30, 95% CI [-.53, -.08], *p* = .008.

*Mean expected side effects* at baseline were low. Non-significant group by time interactions indicated that the groups did not differ, EXPECT vs. SUPPORT: -0.04, 95% CI [-0.16, 0.08], *p* = .51; vs. TAU: -0.11, 95% CI [-0.23, 0.02], *p* = .092. Pre-post comparisons showed no significant change over time in any group.

The *mean expected ability to cope with potential side effects*, which was analyzed for 172 patients who expected at least one of the 44 side effects, was high at baseline. A significant group by time interaction indicated improved coping expectations in EXPECT compared to TAU, but not to SUPPORT, 0.22, 95% CI [0.04, 0.39], *p* = .015; 0.12, 95% CI [-0.05, 0.29], *p* = .15. Pre-post comparisons indicated that coping expectations significantly improved in EXPECT, but not in TAU or SUPPORT, 0.15, 95% CI [0.03, 0.27], *p* = .013; -0.07, 95% CI [-0.20, 0.06], *p* = .30, 0.03, 95% CI [-0.09, 0.15], *p* = .65.

*Treatment control expectations* at baseline were moderately high. A significant group by time interaction indicated that EXPECT developed significantly higher treatment control expectations compared to TAU, but not to SUPPORT, 1.41, 95% CI [0.56, 2.27], *p* = .001; 0.70, 95% CI [-0.14, 1.54], *p* = .10. Pre-post comparisons indicated that treatment control expectations declined in TAU, but did not change in EXPECT or SUPPORT, -1.12, 95% CI [-1.73, -.51], *p* < .001; 0.30, 95% CI [-0.30, 0.89], *p* = .33; -0.40, 95% CI [-1.00, 0.19], *p* = .18.

### Changes in Adherence Intention

Adherence intention at baseline was high (Table 2). EXPECT developed a significantly higher intention to adhere to their AET compared to SUPPORT, and in trend compared to TAU, 0.71, 95% CI [0.09, 1.33], *p* = .024; 0.59, 95% CI [-0.04, 1.22], *p* = .065 (Figure 2). Pre-post comparisons indicated that adherence intention significantly increased in EXPECT, but not in TAU and SUPPORT, 0.61, 95% CI [0.17, 1.04], *p* = .007; 0.01, 95% CI [-0.44, 0.47], *p* = .96; -0.11, 95% CI [-0.54, 0.33], *p* = .63, respectively.

### Patients’ Evaluation of the Intervention

**Figure 3:**
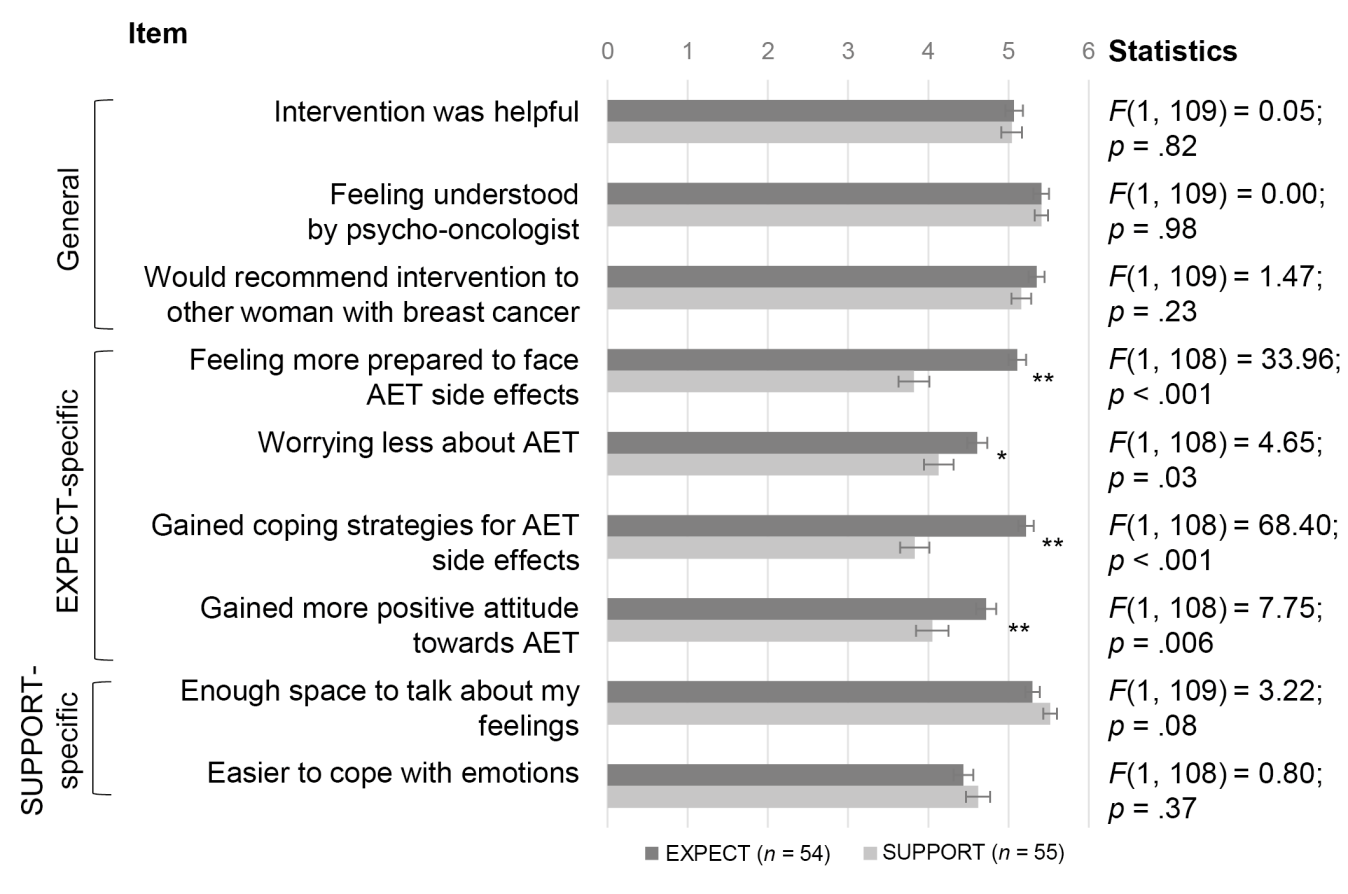
Patients’ Evaluation of EXPECT and SUPPORT Interventions *Note.* General = general satisfaction with the intervention; EXPECT-specific = specific components of expectation management training; SUPPORT-specific = therapeutic components imminent to supportive therapy. Statistics are between-group comparisons (ANOVAs). *p < .05. **p < .01.

The general satisfaction was very high in both groups, while the EXPECT-specific components (e.g., feeling more prepared to face AET side effect) were evaluated more positively in EXPECT (Figure 3). SUPPORT-specific components (e.g., easier to cope with emotions) were evaluated non-significantly better in SUPPORT.

Regarding *adverse events* of the interventions, 14 patients in EXPECT and 13 patients in SUPPORT reported at least one adverse event. In EXPECT, patients reported: organizational issues (4), the number of sessions being too few (4) or too many (1), too much focus on adverse events (2), emotional distress (1), needing more recommendations on coping with side effects (1), and having no need for the intervention (1). In SUPPORT, patients reported: organizational issues (4), too little focus on AET (3), too much focus on possible adverse events (1), the number of sessions being too few (1), needing more recommendations on coping with side effects (1), emotional distress (1), and wish for being asked more questions (1).

Regarding *treatment fidelity*, 39 patients in EXPECT (70.9%, 55 datasets available) practiced their individual protective image developed in the intervention at least once a week. Moreover, at least one booster session was taken up by 52 patients (76.5%) in EXPECT and 51 patients (75%) in SUPPORT.

Regarding *therapeutic alliance*, patients in both groups highly agreed that the intervention had helped them, EXPECT: *M* (*SD*) = 5.70 (0.40); SUPPORT: 5.62 (0.47), and that they felt understood, 5.43 (0.52); 5.30 (0.61). Therapists fully agreed that the intervention might have helped the patient, EXPECT: 5.07 (0.75); SUPPORT: 4.73 (1.09), and that patients felt understood, 5.27 (0.70); 5.32 (0.61). Patient and therapist ratings showed medium correlations across both groups, item help: *r* = .408, *p* < .001; item understanding: *r* = .317, *p* < .001).

## Discussion

This randomized controlled trial investigated whether a brief expectation-focused psychological intervention (EXPECT) optimizes patients’ medication-related expectations before starting AET for breast cancer. In summary, patients’ necessity-concern beliefs about AET were significantly optimized in EXPECT as compared to both TAU and SUPPORT. Expected coping with side effects and expected treatment control were significantly optimized compared to TAU but not to SUPPORT. Expected adherence was significantly optimized compared to SUPPORT but not to TAU. Expected side effects did not change significantly.

As predicted, patients receiving EXPECT developed more positive AET-related expectations compared to both SUPPORT and TAU. In particular, patients in EXPECT increased their necessity beliefs and reduced their concerns, while necessity-concern beliefs remained unchanged in the other groups. This result is highly relevant given that dysfunctional necessity-concern beliefs are associated to poorer medication adherence (Horne et al., 2013), which in turn predicts morbidity and mortality in breast cancer (Hershman et al., 2011). The relevance of these changes is underpinned by the increase in adherence intention compared to SUPPORT and in trend to TAU, which is a good predictor of actual adherence (Manning & Bettencourt, 2011). Accordingly, compared to TAU, patients receiving EXPECT expected to cope better with possible side effects and had higher expectations that AET could control their illness.

To our knowledge, this is the first study investigating expectation change in cancer treatment. Our findings are in line with previous evidence from the PSY-HEART trial targeting expectations prior to cardiac surgery (Laferton et al., 2016; Rief et al., 2017), an RCT addressing illness perceptions after myocardial infarction (Broadbent, Ellis, Thomas, Gamble, & Petrie, 2009), and experimental pain research (Peerdeman et al., 2016). All showed that patients’ expectations can be effectively changed through brief interventions using expectation management, verbal suggestions, imagery, or conditioning. In breast cancer, an acupressure band combined with expectation-enhancing information reduced nausea after chemotherapy in patients with high levels of expected nausea, but the authors did not report expectation change (Roscoe et al., 2010). In our opinion, thoroughly assessing expectation changes is highly relevant to understand how interventions work, and whether postulated etiological mechanisms are actually targeted. While there are effective approaches to support patients in coping with cancer-associated stress, pain, and fatigue (Antoni et al., 2009), few directly address coping with side effects of cancer treatment (Mann et al., 2012) and target patients’ expectations as a relevant etiological factor.

With small to moderate effect sizes, EXPECT was specifically superior to our psychological control condition (SUPPORT) in changing medication beliefs and improving adherence intention. In contrast, changes in coping and treatment control expectations did not significantly differ between EXPECT and SUPPORT. While most effects indicated in the assumed direction, proving superiority to a strong, active control condition like supportive therapy might need larger sample sizes. Thus, EXPECT can be considered effective compared to TAU and partly superior to SUPPORT for some of the expectation measures.

Contrary to our hypothesis, the mean intensity of expected side effects did not change significantly, for which two aspects might be relevant. Firstly, discussing side effects might not actually reduce their expected intensity. Importantly, our study shows that the guided therapeutic attention on side effects is not harmful, as might be feared by physicians and patients. This is in line with studies showing that the assessment of side effect expectations does not increase their occurrence (Colagiuri et al., 2013). However, we will carefully monitor the occurrence of adverse effects in our trial (von Blanckenburg et al., 2013). Secondly, ceiling effects due to low baseline side effect expectations might explain the lack of changes. It is possible that our provision of standardized comprehensive information about AET to all patients already lowered side effect expectations (Heisig, Shedden-Mora, von Blanckenburg, et al., 2015).

With regard to the patients’ evaluation, both interventions were well accepted and perceived as highly helpful, while all EXPECT-specific elements were rated as more achieved in EXPECT. Thus, the interventions can be regarded as specific in targeting the aimed mechanism from the patients’ perspective. Importantly, the therapeutic alliance from both the patients’ and the therapists’ perspective was perceived as very supportive.

Few patients experienced adverse events of the intervention, of which most were of organizational nature. Two patients in EXPECT feared that the focus on possible adverse events might make them more sensitive to actually experiencing them. While there was no overall increase in side effect expectations in our study, these concerns need to be taken seriously and addressed in nocebo-focused expectation management interventions. Taken together, the evaluation shows that both interventions were well accepted and feasible within guideline-based breast cancer care.

### Study Limitations

The results of this RCT need to be interpreted in light of potential limitations. First, while the sample was recruited from four independent sites and resembled a typical early-stage breast cancer sample (Burstein et al., 2014; Murphy et al., 2012), a sample selection bias due to declining participation or non-initiation of AET might limit generalizability. Second, the GASE-expect scales need further psychometric evaluation. Lastly, larger samples might be needed to detect smaller differences between EXPECT and SUPPORT.

### Clinical Implications

In conclusion, this RCT is the first study to show that expectations regarding breast cancer treatment can be effectively changed via a brief psychological intervention. Expectation management proved to be a feasible, well-accepted, effective intervention that was partly superior to the psychological control condition. It could easily be implemented in routine care for women with early-stage breast cancer.

In this study, certain aspects of expectations such as the necessity-concern balance, coping and treatment control expectations seemed more amenable to change. Certainly, more validated assessment methods of patients’ expectations are needed, for which our proposed integrative model of patients’ expectations (Laferton et al., 2017) might provide a framework. Moreover, patients’ expectations result from a dynamic interaction of cognitive processes and experiences with medication intake (Wiech, 2016) and thus might change with the actual experience of AET intake. Therefore, investigating expectation change more systematically seems worthwhile (Heisig, Shedden-Mora, Hidalgo, & Nestoriuc, 2015; Kube, Rief, Gollwitzer, & Glombiewski, 2018).

The long-term effects of these optimized expectations within the PSY-BREAST trial regarding side effect burden, quality of life, and medication adherence (von Blanckenburg et al., 2013) will be reported elsewhere. Moreover, the course of expectations during long-term AET intake and their impact on the above mentioned outcomes will be reported elsewhere. Investigating whether expectations and beliefs can be effectively changed through brief interventions is the first important step towards improving long-term outcomes during AET treatment, and allows for analyzing the effects of expectations changes on clinical outcomes.

## Appendix: Therapist Allegiance

Video ratings of 46 (of 330 available) randomly selected therapy session videos (14%; 24 of EXPECT, 22 of SUPPORT equally selected from the three sessions) were performed by two trained independent raters following a standardized protocol. Ten specific items for EXPECT and SUPPORT assessed objective allegiance on a 1 (‘not present’) to 3 (‘strongly present’) rating-scale (e.g., adherence to manual and structure of sessions, therapeutic attitude). Overall, the mean ratings (with standard deviations in parenthesis) of treatment allegiance in EXPECT and SUPPORT were 2.91 (0.11), and 2.98 (0.05). The percentage in which the two raters fully agreed in their rating of a video was 92% for EXPECT and 99% for SUPPORT.

Subjective allegiance was rated by the therapist after each session on one item scaled from 1 (‘low’) to 4 (‘high’). The mean subjective allegiance ratings in EXPECT and SUPPORT were 3.25 (0.78), and 2.94 (0.33). Thus, therapist subjective and objective allegiance to the respective manuals can be regarded as high.
